# In Situ β-Glucan Fortification of Cereal-Based Matrices by *Pediococcus parvulus* 2.6: Technological Aspects and Prebiotic Potential

**DOI:** 10.3390/ijms18071588

**Published:** 2017-07-21

**Authors:** Adrián Pérez-Ramos, María Luz Mohedano, Paloma López, Giuseppe Spano, Daniela Fiocco, Pasquale Russo, Vittorio Capozzi

**Affiliations:** 1 Centro de Investigaciones Biológicas (CIB), CSIC, Ramiro de Maeztu 9, 28040 Madrid, Spain; apramos@cib.csic.es (A.P.-R.); mmoheda@cib.csic.es (M.L.M.); plg@cib.csic.es (P.L.); 2 Department of Agriculture, Food and Environment Sciences, University of Foggia, Via Napoli 25, 71122 Foggia, Italy; giuseppe.spano@unifg.it (G.S.); vittorio.capozzi@unifg.it (V.C.); 3 Department of Clinical and Experimental Medicine, University of Foggia, Via Pinto 1, 71122 Foggia, Italy; daniela.fiocco@unifg.it; 4 Promis Biotech srl, Via Napoli 25, 71122 Foggia, Italy

**Keywords:** *Pediococcus parvulus*, exopolysaccharides, β-glucans, functional foods, bio-fortification

## Abstract

Bacterial exopolysaccharides produced by lactic acid bacteria are of increasing interest in the food industry, since they might enhance the technological and functional properties of some edible matrices. In this work, *Pediococcus parvulus* 2.6, which produces an *O*2-substituted (1,3)-β-d-glucan exopolysaccharide only synthesised by bacteria, was proposed as a starter culture for the production of three cereal-based fermented foods. The obtained fermented matrices were naturally bio-fortified in microbial β-glucans, and used to investigate the prebiotic potential of the bacterial exopolysaccharide by analysing the impact on the survival of a probiotic *Lactobacillus plantarum* strain under starvation and gastrointestinal simulated conditions. All of the assays were performed by using as control of the *P. parvulus* 2.6’s performance, the isogenic β-glucan non-producing 2.6NR strain. Our results showed a differential capability of *P. parvulus* to ferment the cereal flours. During the fermentation step, the β-glucans produced were specifically quantified and their concentration correlated with an increased viscosity of the products. The survival of the model probiotic *L. plantarum* WCFS1 was improved by the presence of the bacterial β-glucans in oat and rice fermented foods under starvation conditions. The probiotic bacteria showed a significantly higher viability when submitted to a simulated intestinal stress in the oat matrix fermented by the 2.6 strain. Therefore, the cereal flours were a suitable substrate for in situ bio-fortification with the bacterial β-glucan, and these matrices could be used as carriers to enhance the beneficial properties of probiotic bacteria.

## 1. Introduction

Bacterial exopolysaccharides (EPS) are extracellular polymers which can be tightly adhered (i.e., capsular), loosely associated to the microbial cell surface, or released into the environment [1,2,3,4]. Lactic acid bacteria (LAB) are a heterogeneous group of Gram-positive prokaryotes characterised by a long history of safe use by humans, and are of outstanding relevance for the production of fermented food and beverages [5,6]. Several LAB strains are able to produce EPS, either heteropolysaccharides or homopolysaccharides, mainly including β-d-glucans, α-d-glucans, levans, and β-d-galactans [2]. Based on their functional and technological properties, bacterial EPS are of interest for new developments in food, biomedical, and pharmaceutical applications [7,8]. It is well known that EPS can act as natural viscosifiers and thickening agents, thus suggesting the use of EPS-producing LAB as starter cultures to enhance the texture and mouthfeel properties of some foods [9,10,11]. These biopolymers have also been related with the mechanisms of cellular recognition and biofilm formation, thus modulating the probiotic colonization of the gut ecosystem [12,13]. Moreover, EPS from LAB are good candidates for prebiotics, since they are considered fermentable substrates by the intestinal microbiota [14]. Furthermore, the consumption of bacterial EPS seems to provide health benefits including anti-tumour, immunomodulating, or antiviral effects [15,16,17,18]. Finally, EPS-produced by LAB can protect the producing organisms against the conditions associated with food processing and help with passage through the oro-gastrointestinal tract, thus encouraging the colonization of the gut environment [19].

Among LAB, *Pediococcus parvulus* strains were found to be the main biological agents of ropiness in cider and wine [20,21], due to their ability to produce an EPS with a primary structure consisting of a trisaccharide repeating unit with two (1,3)-β linked residues in the main chain, one of which is substituted in position 2 by a terminal glucose residue (*O*2-substituted (1,3)-β-d-glucan). Studies performed with the *P. parvulus* 2.6 strain isolated from cider demonstrated that this β-glucan is synthesised by the glycosyltransferase (GTF), whose coding *gtf* gene is carried in the pPP2 plasmid [22,23,24], and curing of the plasmid resulted in the generation of the non-ropy strain 2.6NR [20]. Although detrimental for the production of alcoholic beverages, the ropy phenotype of EPS-producing *P. parvulus* strains has been proposed as a beneficial feature for the production of functional foods [25]. The complete draft genome of *P. parvulus* 2.6 was recently announced [26], and in recent years its *O*2-substituted (1,3)-β-d-glucan has been well characterised [27,28]. The presence of this EPS influences some probiotic features of *P. parvulus* 2.6, including the enhancement of its adhesion to human enterocyte cell lines, and the reduction of the production of inflammation-related cytokines by polarized macrophages [29,30]. The purified β-glucan from *P. parvulus* 2.6 was also able to induce macrophages’ activation with anti-inflammatory effects [31], and to stimulate the growth of probiotic LAB and their adhesion to CaCo-2 cells, thus suggesting its employment as a prebiotic and immunomodulator [32]. Furthermore, the health-promoting effects of the *O*2-substituted (1,3)-β-d-glucan from *P. parvulus* 2.6 have led to encouraging results also when tested in in vivo models. Thus, in human trials, the administration of an oat product fermented by *P. parvulus* 2.6 showed a significant bifidogenic effect having a beneficial effect on the blood cholesterol level [33], which has not been detected in bacteria producing other EPS. Recently, Lindström et al. investigated the influence of a diet supplemented with *P. parvulus* 2.6 in the digestive tract of mice [34]. These authors found that some responses, including hypocholesterolemic potential, could be modulated in a different way by live bacteria rather than by their purified β-glucans [35]. Moreover, the heterologous expression of the *gtf* gene of *P. parvulus* 2.6 in a recombinant *Lactobacillus paracasei* resulted in the modulation of lipid metabolism in a mouse model of atherosclerosis [36]. Therefore, the usage of *P. parvulus* 2.6 has been suggested for the preparation of functional foods [37,38], and the β-glucan production by its heterologous expression has been proposed for potential use as a food additive [23].

In this work, we have evaluated *P. parvulus* 2.6 for the production of cereal-based fermented foods that are naturally bio-fortified in microbial β-glucans, in order to improve the technological and functional features of the products. Moreover, in order to investigate the prebiotic potential of the in situ synthesised EPS, the food matrices were tested as probiotic carriers by analysing the impact on the survival of the probiotic *Lactobacillus plantarum* WCFS1 under starvation and in response to simulated gastrointestinal conditions.

## 2. Results

### 2.1. Production of Cereal-Based Fermented Foods

**Figure 1:**
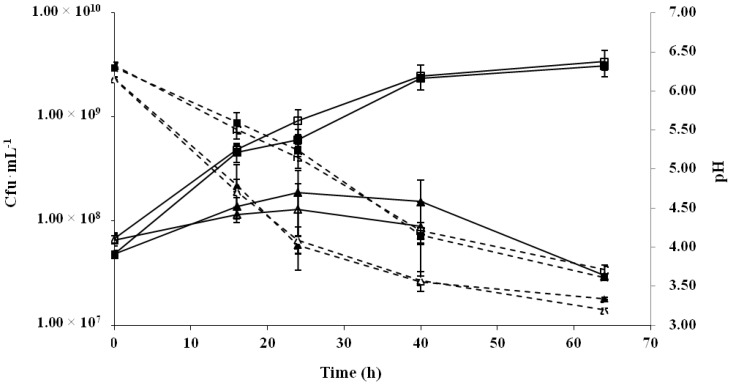
Fermentation of oat (square) and rice (triangle) matrices by *Pediococcus parvulus* 2.6 (black symbols) and 2.6NR (white symbols) strains. The evolution of bacterial viability (continuous line) and the pH of the matrices (dashed line) were monitored over a 64 h period.

In the present work, the *O*2-substituted (1,3)-β-d-glucan-producing *Pediococcus parvulus* 2.6 was investigated for its ability to ferment and to produce β-glucan in a cereal-based matrix in comparison with its isogenic EPS-non-producing 2.6NR strain. With this aim, three different cereal-based fermented products were obtained from the flours of oat, rice, or barley, according to the protocol reported by Russo et al. [39]. After a thermal treatment at 95 °C for 15 min, to encourage the gelatinization of the cereal matrices, the environmental microbial contamination was maintained at lower than 100 cfu·mL^−1^. Then, fermentations were carried out by inoculating with either the 2.6 or 2.6NR strains at a concentration of about 5 × 10^7^ cfu·mL^−1^. In order to optimise the conditions for the fermentations, the viability of the pediococci, the pH of the food matrices, and the microbial β-glucan production were monitored during 64 h, as reported in Figure 1 and Table 1.

The two strains failed to grow during the barley flour fermentations. In fact, the microbial viability was below 10^5^ cfu·mL^−1^ after 24 h, and the pH was almost stable, ranging from 5.5 to about 5.0 at the beginning and at the end of the fermentation step, respectively. Moreover, no microbial EPS production was detected in the barley-based samples inoculated with *P. parvulus* 2.6 (Table 1). Therefore, the occurrence of antimicrobial compounds in the barley matrix was examined, but no inhibition halo was detected by spotting the food product on MRS plates, supporting the hypothesis that the tested strains were unable to ferment the barley flour. For these reasons, a barley-based product was discarded in further investigations.

In the oat and rice matrices, both strains showed the same growth rate and pattern as well as identical acidification (Figure 1). In general, oat flour seems to be an excellent substrate to support the growth of *P. parvulus*, since the viability increased from 5 × 10^7^ cfu·mL^−1^ up to about 3 × 10^9^ cfu·mL^−1^ after 64 h of fermentation. Nonetheless, the high microbial concentration was not associated with a fast acidification of the food, since the pH decreased to 4.2 and 3.6 after 40 and 64 h of fermentation, respectively (Figure 1). By contrast, during the fermentation of the rice-based product, the viability of both strains was almost constant during the first 40 h, and then slightly declined, whereas a faster acidification of the matrix was observed; after only 24 h of fermentation the pH was about 4.0 and then further decreased to 3.2 (Figure 1). As shown in Table 1, the EPS production was about threefold higher during the fermentation of oat- than rice-based foods at all the experimental times tested, while, as expected, no EPS was detected in the food fermented by *P. parvulus* 2.6NR.

In order to achieve the best combination of fermentation time, acidification rate, and enrichment of the EPS content, a fermentation of 40 h was carried out for the production of oat- and rice-based foods. After this time, the product obtained by 2.6NR fermentation showed a looser consistency than that fermented by the 2.6 strain, suggesting that microbial EPS improved the rheological features of these foods. Therefore, the apparent viscosity was determined and the corresponding results are reported in Table 2. As expected, the samples inoculated with *P. parvulus* 2.6 were always more viscous than the products fermented by the 2.6NR mutant. Differences in viscosity were also observed between the two matrices, with that of the rice product fermented by 2.6NR strain being about twofold higher than that of the corresponding oat sample. This could be due to the fact that the determination of viscosity is not very accurate for samples consisting of slurry-containing particles. Nonetheless, when the cereals were fermented by the EPS-producing strain, the viscosity was higher in the oat-based product, in agreement with the larger amount of β-glucans produced in this matrix (Table 2).

### 2.2. Formulation of Probiotic Cereal-Based Fermented Substrates

With the aim to produce a suitable substrate for the growth of probiotic bacteria, the fermented products were submitted to a thermal treatment (80 °C for 20 min) in order to eliminate any vegetative cell of *P. parvulus* and inoculated with *L. plantarum* WCFS1 as a model probiotic [40,41].

### 2.3. Prebiotic Potential of the P. parvulus 2.6 β-Glucans in Probiotic Cereal-Based Fermented Products

**Figure 2:**
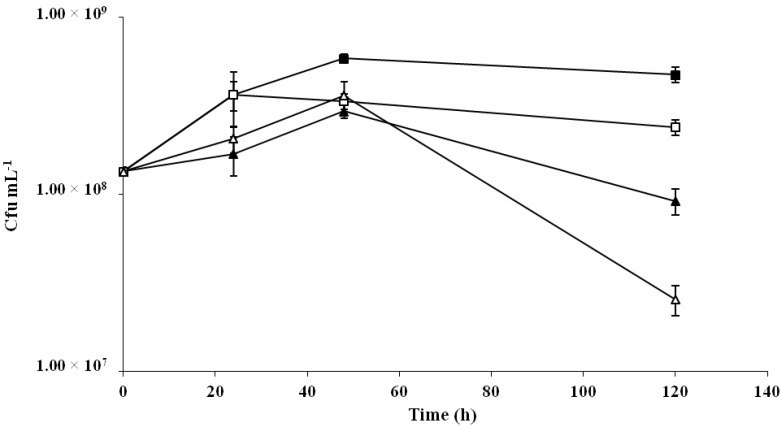
Analysis of prebiotic potential of the *O*2-substituted (1,3)-β-d-glucan. The viability of *Lactobacillus plantarum* WCFS1 was monitored over a 5-day period in oat (square) and rice (triangle) food matrices, previously fermented for 40 h by *P. parvulus* 2.6 (black symbols) or 2.6NR (white symbols) strains.

In order to evaluate the prebiotic potential of the microbial EPS, an aliquot of the fermented cereal product was incubated at 37 °C, and the viability of *L. plantarum* WCFS1 was monitored during 5 days. The control samples were the foods fermented by the 2.6NR strain. A general increasing of the probiotic concentration was observed in the oat-based food during the experimental period. However, in the oat samples fermented by the β-glucan producer *P. parvulus* 2.6, the viability of *L. plantarum* WCFS1 after 48 h was about twofold higher than that detected in the corresponding 2.6NR fermented samples, and this difference was still observed after 5 days of incubation at 37 °C (Figure 2). In the rice-based product, a reduction of the viability of *L. plantarum* WCFS1 was detected during the incubation time, which could be attributed to the lower pH achieved in this food matrix. Nonetheless, after 5 days the probiotic viability was around 10-fold higher in the food fermented by the 2.6 strain (Figure 2).

To investigate the relationship between the improved growth of *L. plantarum* WCFS1 and the occurrence of microbial EPS in the food matrix, the concentration of the *P. parvulus* 2.6’s β-glucan was quantified by the specific immunological method. The results showed the absence of any significant difference in the amount of EPS in the food matrices before the inoculum of the probiotic bacteria and at the end of the incubation (Table 3), thus indicating that *L. plantarum* WCFS1 had not metabolized the microbial β-glucans.

### 2.4. Tolerance to Simulated Gastrointestinal Conditions Using Cereal Based Fermented Products as Carrier Matrix

**Figure 3:**
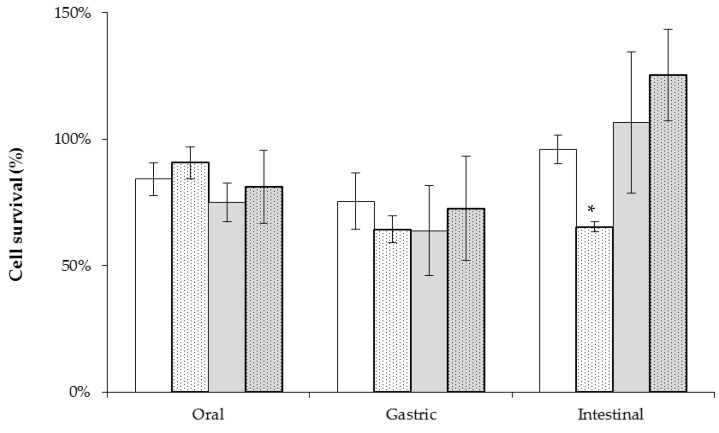
Analysis of protective influence of the *O*2-substituted (1,3)-β-d-glucan against digestive tract stresses. Cell survival of *L. plantarum* WCFS1 when exposed to simulated oro-gastrointestinal conditions using as carrier matrix an oat (white bars) or rice (grey bars) product fermented by *P. parvulus* 2.6R (full bars) or 2.6NR (dotted bars). The percentage of survival was relative to that of unstressed control samples. * *p* < 0.01 compared with the other experimental conditions.

Prior to the test of resistance to gastrointestinal stresses, *L. plantarum* WCFS1 was included in the matrices fermented by the pediococci and subjected to cold storage at 4 °C for 21 days. The viability of the probiotic *L. plantarum* strain was almost stable, which is in accordance with previous findings, using a similar food product [39]. Then, *L. plantarum* WCFS1, included in the different matrices, was submitted to a simplified model mimicking the transit throughout the oro-gastrointestinal tract. As depicted in Figure 3, a reduction in the viability of *L. plantarum* WCFS1 (ranging from 10% to 25%) was observed after exposure to the oral stress. The viability of the probiotic bacteria decreased further (approximately 10%) when submitted to the simulated gastric conditions. By contrast, after 1 h of incubation under conditions reproducing the intestinal environment, the probiotic bacteria recovered its viability, especially in the rice-based products. The survival of the probiotic strain remained constant (about 65%) under gut conditions only when it was carried by an oat-based matrix previously fermented by the *P. parvulus* 2.6NR, i.e., not containing microbial β-glucans. Conversely, in the oat products containing β-glucan synthesised by *P. parvulus* 2.6, the survival rate of *L. plantarum* WCFS1 was more than 95%.

## 3. Discussion

In the present work, the ropy strain *P. parvulus* 2.6, which produces the immunomodulatory EPS *O*2-substituted (1,3)-β-d-glucan, was investigated for its ability to ferment different cereal matrices in order to obtain novel functional food products. To date, *P. parvulus* 2.6 has been proposed to formulate oat-based fermented foods [37], yogurt, orange juice, or juice-milk beverages [38]. In recent years, innovative fermented cereal-based products have emerged as significant sources of functional ingredients with a value as alternative vehicles for the delivery of probiotic bacteria [39,42,43]. Therefore, in this work, three different cereal-based foods were obtained by the fermentation of oat, barley, and rice flours. These cereals were selected in order to analyse the technological and prebiotic potential of the microbial *O*2-substituted (1,3)-β-d-glucan in food matrices that are natural sources (oat and barley) or not (rice) of vegetable β-glucans.

*P. parvulus* 2.6 was unable to ferment barley flour, probably due to the carbohydrate composition of this matrix. This accords with the previous finding [44] that only minor growth and no significant effect on viscosity were detected in a barley β-glucan suspension fermented by *P. parvulus* 2.6. In this regard, it was reported that the cereal source strongly affects the kinetics of fermentation and of EPS production of *Weissella cibaria* MG1 [45]. In contrast, oat and rice were fermented by *P. parvulus* 2.6 and its isogenic 2.6NR mutant, although with different kinetics. In fact, a faster acidification and a lower final pH were detected in the rice flour fermentation compared to the oat, which could partially explain the observed higher microbial viability in the oat matrix. This observation could be attributable to different buffering properties of the flours, as suggested by Wolter et al., who found similar results among wheat sourdough or buckwheat, quinoa, and teff flour fermentations [45].

In the present work, for the first time, a specific immunological method previously developed by us [24] was successfully used to detect and quantify bacterial *O*2-substituted (1,3)-β-d-glucan in food matrices. Compared with other methods used for EPS isolation and quantification (as recently reviewed [46]), the greatest advantage of this technique was the direct detection and quantification of this EPS in a complex background containing other polysaccharides, and even in the presence of the linear β-d-glucan with (1,3) and (1,4) linkages from oat. As expected, the bacterial β-glucan concentration increased with the fermentation time. Moreover, a higher production of EPS was detected in the oat than in the rice, probably depending on the higher viability of *P. parvulus* 2.6 in the oat matrix and/or on a different availability of carbohydrate sources [47].

An objective of this study was to optimise the fermentation conditions of different cereal-based flours in a way this is compatible with the in situ enrichment of the food products with the *P. parvulus* 2.6 β-glucan, in an attempt to harmonise technological and functional parameters. Therefore, it was considered that the higher EPS concentration detected after 64 h was insufficient to justify an increase of one extra day of the fermentation process. Nevertheless, after 40 h of fermentation in oat and rice products, the EPS concentration was respectively higher or similar to that in the functional food administered by Mårtensson et al. [33].

It is well known that bacterial EPS can improve the rheological features of some foods [8]. Therefore, according to the eco-friendly concept of food bio-fortification by bacterial fermentation, the employment of EPS-producing LAB for the in situ production of β-glucans was suggested as an alternative strategy to replace the addition of hydrocolloid additives as texturisers in vegetable containing products [48,49]. As expected, we found that the β-glucan production in the fermented oat and rice flours was associated with the enhanced viscosity of both products, which was more evident during oat fermentation. As reported by Velasco et al., the rheological properties of the EPS produced by *P. parvulus* 2.6 were influenced by culture medium or sugar source [28], suggesting that the food matrix could drive the production of β-glucan with different physicochemical features. In agreement with these observations, an increased viscosity was obtained by *P. parvulus* 2.6 oat fermentation [37], but the exposure to this bacterial strain had no influence on the rheological properties of yogurt, orange juice, and juice-milk beverages [38].

In one of our previous works, the addition of purified β-glucan from *P. parvulus* 2.6 to the culture media improved the growth of probiotic lactobacilli (LAB) and their adhesion to human enterocyte-like cells, suggesting a prebiotic potential of this microbial EPS [32]. Accordingly, here we observed that the occurrence of the *P. parvulus* 2.6 β-glucan synthesised in situ in both cereal-based fermented foods was accompanied by a higher survival rate of a probiotic LAB, i.e., *L. plantarum* WCFS1, which was later inoculated in the fermented flour matrix. In particular, this effect was more apparent after longer fermentation periods, supporting the hypothesis that this EPS, like others, could be involved in the modulation of the growth when probiotics are exposed to critical stress conditions [19]. The concentration of the microbial β-glucan was constant in the food products throughout 5 days of incubation with *L. plantarum* WCFS1, indicating that EPS could play a role in increasing the tolerance to the food abiotic stress, rather than being metabolised as an energy source [50], although we cannot rule out that the growth of the probiotic bacteria was not correlated with the presence of the EPS. Nevertheless, the lack of catabolism of the *O*2-substituted (1,3)-β-d-glucan in the presence of *L. plantarum* WCFS1 could be advantageous for the development of a functional symbiotic food fermented with *P. parvulus* 2.6 and supplemented with a probiotic, since previous results indicate that the pediococcal EPS, in addition to be a prebiotic, could have an anti-inflammatory as well as hypocholesteronemic effect [31,32,33].

Lysozyme resistance was recently correlated with β-glucan accumulation around the cell of the ropy strain *P. parvulus* IOEB 8801 [51], and microbial EPS have been suggested to contribute to the bacterial tolerance to bile/acid environments [52,53,54]. However, the β-glucan produced by *P. parvulus* 2.6 did not increase the survival of this bacterium in a simulated human digestive tract model [29]. By contrast, the heterologous expression of the *P. parvulus* 2.6 *gft* gene in a recombinant *Lactobacillus paracasei* was associated with significantly increased protection from gastrointestinal stresses [55]. In the present study, *L. plantarum* WCFS1 showed a significant higher viability to simulated intestinal stress when it was included in the oat matrix fermented by the 2.6 strain, hence supporting that the β-glucan EPS might exert a protective function. As the β-glucan content of the oat product was about threefold higher than in the fermented rice flour, it is possible to argue a presumptive protective effect of this microbial EPS to certain stress conditions faced in the gut environment [56]. However, the effect of the food matrix [38,57] rather than the occurrence of microbial β-glucans on the modulation of the resistance to gastrointestinal conditions should be the matter of further investigations.

## 4. Materials and Methods

### 4.1. Microbial Strains and Growth Conditions

The EPS-producing strain *Pediococcus parvulus* 2.6 (formerly *Pediococcus damnosus* 2.6) [22] isolated from a ropy cider and its isogenic EPS-non-producing strain *P. parvulus* 2.6NR, cured of the pPP2 plasmid of the 2.6 strain by chemical mutagenesis [20], were used as the starters for the cereal flours’ fermentation. *Lactobacillus plantarum* WCFS1 was used as a model probiotic bacteria [41]. All of the strains were routinely grown in MRS broth (Oxoid, Basingstoke, UK) at 30 °C for *Pediococcus* strains and at 37 °C for the *Lactobacillus* strain.

### 4.2. Fermented Cereal-Based Foods

A dry ingredient to generate Liquid Whole Oat, obtained from milled whole oat grains without alteration, was provided by Glucanova (Lund, Sweden), while barley and rice flours were acquired by commercial brands. Three cereal-based foods were obtained by fermenting the flour of oat, rice, and barley according to the method previously reported by Russo et al. [39]. Briefly, three mixtures (100 mL each) containing the different cereal flours (18% *w*/*v*) and distilled water (82% *v*/*v*) were prepared in sterile plastic containers with a screwcap. Then, the food matrices were heated to 95 °C for 10 min, and shaken manually every 2 min in a water bath. Then, the food matrices were inoculated with late exponential phase cultures of *P. parvulus* (approximately 5 × 10^7^ cfu·mL^−1^), previously sedimented by centrifugation (8.000 rpm, 10 min), and washed with sterile saline solution (0.86% NaCl, *w*/*v*). The fermentative step was carried out at 30 °C for 64 h. Cell viability, pH variation, β-glucan production, and viscosity were monitored during the fermentation. Three independent experiments were performed in duplicate.

### 4.3. Inhibition Assay of the Barley Matrix

Plates of soft MRS agar were inoculated with either *P. parvulus* 2.6 or 2.6NR at exponential phase (1:100 *v*/*v*), and 10 μL of a cereal-based fermented product from the barley matrix were spotted at the centre of the plates. The occurrence of antimicrobial compounds in the food matrix was determined by the halo of inhibition around the spot.

### 4.4. Viscosity of Cereal-Based Products Fermented by P. parvulus 2.6

Rheological measurements of the fermented cereal products were performed with a rotational Brookfield DV-II + Pro viscometer (Brookfield Engineering, Harlow, UK), and the results analysed with RheocalcT 1.1.13 software (Brookfield Engineering). For the analysis, approximately 50 mL of each sample were placed in the concentric cylindrical cup, and the spindle n° 3 was used to apply shear rates of 40 rpm for 30 s at room temperature. The viscosity was expressed in centipoise (cP).

### 4.5. Quantification of the β-Glucan Produced by the P. parvulus 2.6 Strain

The capsule of *Streptococcus pneumoniae* serotype 37 is a branched β-d-glucan almost identical to the *O*2-substituted (1,3)-β-d-glucan synthesised by *P. parvulus* 2.6. Therefore, we previously developed a competition (ELISA) method for the quantification of the pediococcal EPS based on *S. pneumoniae* serotype 37 antibodies [24], and in this work we have used this method for the *O*2-substituted (1,3)-β-d-glucan quantification in food matrices with the following modifications. The ELISA assay was performed in 96-well Nunc Maxisorpmicrotitre plates (Thermo Fisher Scientific, Waltham, MA, USA). The *P. parvulus* 2.6R EPS (6.5 μg per well), purified as previously described [58], was immobilized in each well as well as various concentrations of the fermented products as competitor for binding to the primary antibody (dilution 1:800 of anti-serotype 37, Statens Serum Institute, Copenhagen, Denmark). When necessary, the samples were diluted with PBS at pH 7.0. Testing of the non-fermented matrices did not reveal any background due to the cereal β-glucans.

### 4.6. Formulation of Probiotic Cereal-Based Fermented Foods

*L. plantarum* WCFS1 was used as a model micro-organism for the formulation of probiotic cereal-based fermented foods. Samples of the products (50 mL) fermented by either *P. parvulus* 2.6 or 2.6NR obtained as reported above were submitted to a thermal treatment of 20 min at 80 °C to eliminate microbial vegetative cells. For the inoculum, *L. plantarum* WCFS1 at late exponential phase was recovered by centrifugation (5000 rpm, 10 min), washed once in sterile saline solution, and finally added to the food to obtain a final concentration of about 1 × 10^8^ cfu·mL^−1^. Then, the products were divided into two aliquots, each of 25 mL, and submitted to further assays.

### 4.7. Prebiotic Potential of Bacterial β-Glucans in Cereal-Based Fermented Foods

With the aim of evaluating the prebiotic potential of the EPS produced by *P. parvulus* 2.6 in the food matrix, one aliquot of the samples prepared as described above was incubated at 37 °C for 5 days to encourage any fermentative activity of *L. plantarum* WCFS1 under starvation conditions. The viability of *L. plantarum* WCFS1 was monitored after 24, 48, and 120 h of incubation at 37 °C. The microbial β-glucan content was measured at the beginning and at the end of the fermentation. The results were compared with the growth of *L. plantarum* WCFS1 in products fermented by *P. parvulus* 2.6NR. Two independent experiments for each assay were performed in duplicate.

### 4.8. Tolerance to Gastrointestinal Conditions Using Cereal-Based Fermented Products as Carrier Matrix

In order to assay the probiotic potential of the fermented products, the second aliquot of the samples was stored at 4 °C for 21 days. The viability of *L. plantarum* WCFS1 was monitored after 0, 7, 14, and 21 days of cold storage by plating appropriate dilutions on MRS agar and subsequent cfu counting. Two independent replicates of each sample were performed.

After 21 days of cold storage, the bacterial cells were exposed to an in vitro model mimicking the gastrointestinal transit, adapted from Bove et al. [59]. First, samples were subjected to an oral stress by adding 150 mg·L^−1^ of lysozyme, adjusting the pH to 6.0, and incubating for 5 min at 37 °C. The gastric conditions were simulated by reducing the pH to 3.0 in the presence of 3 g·L^−1^ of pepsin, and incubating the samples at 37 °C for 30 min with shaking. Finally, the intestinal environment was mimicked by increasing the pH value to 6.5 and adding 1 g·L^−1^ of pancreatin and 3 g·L^−1^ of bile salts. The samples were incubated for 60 min at 37 °C with shaking. Bacterial survival was determined by plate counting at each step, and represented as percentage of survival relative to the unstressed control. The same assays were performed by diluting the food matrix 1:10 in sterile saline solution. Two independent experiments for each trial were carried out in duplicate.

### 4.9. Statistical Analysis

For the analysis of the protective influence of the *O*2-substituted (1,3)-β-d-glucan against digestive tract stresses, the data were subjected to a one-way analysis of variance (ANOVA) by using the statistical software Past 3.0. Tukey’s test was employed to determine the significant differences between the means in a pairwise comparison at *p* ≤ 0.05.

## 5. Conclusions

In this study, a β-glucan producing strain of *P. parvulus* was tested for its potential use in the development of novel cereal-based functional foods. First, our results showed that the fermentation process is strongly dependent on the raw material. However, from a technological point of view, the in situ enrichment of the food with this microbial EPS always provided an improvement of the rheological quality of the product. Moreover, the occurrence of *P. parvulus* 2.6 β-glucan seems to increase the tolerance of a model probiotic strain to starvation stress conditions, typical of the food fermentation environment, and to confer survival advantages in the host’s intestinal tract. These findings suggest that the proposed functional products could be used as vehicle matrices to increase the performances of probiotic bacteria. Therefore, the in situ bio-fortification of fermented cereal represents a promising strategy to vehicle this microbial EPS and further efforts should be encouraged to design and to optimise the production of new food products as carriers of these molecules. Although several studies have focused on discovering the beneficial health effects of the *O*2-substituted (1,3)-β-d-glucan from *P. parvulus* 2.6 by using both in vitro and in vivo approaches [31,33,34,35], it is worth emphasising that different effects were observed in the gut microbiota composition of mice fed with a diet supplemented with purified EPS or live EPS-producing *P. parvulus* 2.6 [35]. Therefore, further investigations should be performed to determine in vivo the incidence of a synergistic effect attributable to the simultaneous administration of this microbial β-glucan and probiotic bacterium.

## Figures and Tables

**Table 1:** Quantification of *O*2-substituted (1,3)-β-d-glucan (mg·L^−1^) production in oat, rice, and barley flours fermented by *P. parvulus* 2.6. Three independent experiments were performed in duplicate.

Time (h)	Oat	Rice	Barley
16	109 ± 17.9	34.2 ± 13.5	22.8 ± 2.8
24	139.7 ± 40.8	58.8 ± 14.0	21.3 ± 2.0
40	341.7 ± 44.2	129.6 ± 31.0	26.7 ± 9.8
64	659.4 ± 45.1	164.7 ± 52.3	24.9 ± 5.3

**Table 2:** Viscosity (cP) determined at 40 rpm for 30 s of the oat and rice products fermented for 40 h by *P. parvulus* 2.6 or 2.6NR strains. Three independent experiments were performed in duplicate.

Strain	Oat	Rice
*P. parvulus* 2.6	5407 ± 21	4187 ± 108
*P. parvulus* 2.6NR	1810 ± 248	3523 ± 297
**Ratio**	2.99	1.19

**Table 3:** *O*2-substituted (1,3)-β-d-glucan (mg·L^−1^) production in oat and rice products fermented 40 h by *P. parvulus* 2.6 submitted to a thermal treatment, and subsequently inoculated with *L. plantarum* WCFS1 (time 0) and after 5 days incubation at 37 °C. Three independent experiments were performed in duplicate.

Time (h)	Oat	Rice
0	422.7 ± 16	81.6 ± 5.7
120	437.5 ± 40.8	96.4 ± 27.7

## Abbreviations

EPS	Exopolysaccharides
LAB	Lactic acid bacteria
